# Efficacy of an additional flap operation in bladder-preserving surgery with radical prostatectomy and cystourethral anastomosis for rectal cancer involving the prostate

**DOI:** 10.1007/s00595-017-1484-z

**Published:** 2017-03-04

**Authors:** Keita Noguchi, Yuji Nishizawa, Yoshinobu Komai, Yasuyuki Sakai, Akihiro Kobayasi, Masaaki Ito, Norio Saito

**Affiliations:** 1 Department of Colorectal and Pelvic Surgery, National Cancer Center Hospital East, 6-5-1, Kashiwanoha, Kashiwa-City, Chiba 277-8577 Japan; 2 Department of Urology, National Cancer Center Hospital East, Kashiwa, Japan

**Keywords:** Colorectal cancer, Total pelvic excision, Bladder-preserving surgery, Additional flap operation

## Abstract

**Purpose:**

Sphincter-preserving operations performed with bladder-preserving surgery and a cystourethral anastomosis (CUA) do not require a urinary stoma, but leakage from the CUA may develop. The aim of this study was to evaluate the efficacy of performing an additional flap operation.

**Methods:**

The subjects were 39 patients who underwent bladder-preserving surgery for advanced rectal cancer involving the prostate, between 2001 and 2015.32 of whom had a CUA and one of whom had a neobladder. Five of these 32 patients underwent an ileal flap operation, 2 underwent an omental flap operation, and 3 underwent an operation using both flaps.

**Results:**

Leakage developed in 3 (30%) of the 10 patients who underwent additional flap operations, but in 14 (60.9%) of the 23 patients who did not undergo a flap operation. The mean periods of catheterization for the patients who suffered leakage were 31 weeks (8–108 weeks) in those without a flap and 16 weeks (8–20 weeks) in those with a flap. Four (33.3%) of the 12 patients with leakage after surgery without a flap had a period of urinary catheterization >30 weeks, and 2 (16.7%) had leakage of CTCAE grade 3. There were no cases of leakage after flap surgery.

**Conclusion:**

An additional flap operation may decrease the risk of leakage from a CUA.

## Introduction

Abdominoperineal resection (APR) is the standard surgery for locally advanced rectal cancer located within 5 cm of the anal verge [1]. With tumor involvement of the base or trigone of the bladder and prostate, total pelvic exenteration (TPE) may be required to achieve negative margins [2]. These patients often require double stomas: one for urinary diversion, such as an ileal conduit; and another for fecal diversion, such as a sigmoid colostomy. However, this procedure compromises quality of life severely. Recent advances in sphincter-preserving operations (SPOs) including intersphincteric resection (ISR) and Ultra-low anterior resection (U-LAR) for very low rectal cancer have allowed Coloanal Anastomosis (CAA) or coloanal canal anastomosis to be performed without adverse effects on outcome [3–9]. Neobladder construction has also become a standard procedure following cystoprostatectomy for invasive bladder cancer [10, 11]. Conversely, en bloc radical prostatectomy is an option for selected patients who would otherwise need TPE for locally advanced rectal cancer involving the prostate [12–15]. Bladder-preserving surgery with cysto-urethral anastomosis (CUA) allows for voiding via the urethra with urinary continence. Collectively, these advances may improve post operative quality of life for patients with locally advanced rectal cancer requiring TPE by enabling surgery to be performed without a stoma or with only a single stoma [16–20]. We have explored these approaches in patients with locally advanced primary or recurrent rectal cancer at our institute since 2001. As leakage from a CUA is a concern associated with this surgery, we conducted a prospective study to evaluate the effectiveness of additional flap surgery for reducing this leakage in bladder-preserving surgery for locally advanced primary rectal cancer.

## Methods

### Patients

Between January, 2001 and January, 2015, 39 patients with locally advanced primary and recurrent rectal cancer with clinical involvement of the prostate underwent extended colorectal resection, combined with radical prostatectomy, at the National Cancer Center Hospital East. All 39 patients were originally considered to be candidates for TPE or ISR with radical cystoprostatectomy. However, ultimately, the urinary bladder was preserved in 38 patients and a neobladder was reconstructed in one patient, to avoid TPE and radical cystoprostatectomy. Four patients agreed to undergo preoperative radiochemotherapy based on our previous protocol [6], although this is not standard protocol for resectable rectal cancer in Japan. The rectal tumor was staged using the 7th UICC TNM staging system.

Table 1 lists the age, tumor grade, and clinical tumor stage of the 39 patients. Four of the patients had cT1b-3 tumors with synchronous primary prostatic cancer and as all these patients underwent CUA, they were included in the study. The mean age was 63 years (range 26–76 years) and the mean distance from the tumor to anal verge was 3.5 cm (range 0–6.5 cm). To measure the distance from the rectal tumor to the anal verge, rigid proctoscopy with measurement and/or digital examination was used. Preoperative staging was done using a transanal digital examination, computed tomography (CT), magnetic resonance imaging (MRI), endoscopic ultrasonography, colonoscopy, and barium enema. Position emission tomography was also performed preoperatively to exclude widespread metastatic disease.

**Table 1:** Clinical characteristic of the patients

	Primary, *n* = 34	Recurrence, *n* = 5
Mean age, years (range)	65 (43–76)	55 (43–63)
Mean distance from AV, cm (range)	3.5 (0-6.5)	Unknown
Depth of invasion (clinical)	cT4:20 (58.8%)	
	cT3:12 (35.3%)	
	cT1b:1 (2.9%)	
	Unknown:1 (2.9%)	
Involved organs	Prostate (SV,SU,B,L):18 (90%) SV:1 (5%) B + U:1 (5%)	P:5 (100%)
Node involvement (clinical)	cN(+):17	cN(+):0

*AV* anal verge, *P* prostate, *SV* seminal vesicle, *SU* urethral sphincter muscle, *B* bladder, *U* ureter, *L* levator muscle

All patients had a localized rectal tumor involving the prostate and seminal vesicle, or combined with carcinoma of the prostate, without evident distant metastases preoperatively. All resected specimens were examined clinicopathologically and involvement of adjacent organs and surgical margins, perioperative morbidity and mortality, locoregional control, overall survival (OS), and disease-free survival (DFS) were investigated. Informed consent was obtained from all patients and approval was given by the institutional review board. The study was performed in accordance with the Helsinki Declaration of 1975 and 1983.

### Indications and surgical procedure

Bladder-sparing extended en bloc rectal resection combined with radical prostatectomy was considered if the tumor was adhered to the prostate or bladder, or if an adequate margin between the tumor and these organs seemed impossible to achieve, based on magnetic resonance imaging (MRI) and computed tomography (CT) findings. Patients undergoing this surgical procedure had a residual urinary bladder with available capacity of 50 ml and the possibility for CUA. Distant metastasis, wide-ranging involvement of the urinary bladder, prostate, and marked pelvic lymph node metastasis were generally considered contraindications for this operation, since preservation of the available urinary bladder was impossible in patients with wide-ranging involvement of the urinary bladder. Those patients underwent TPE.

**Fig. 1:**
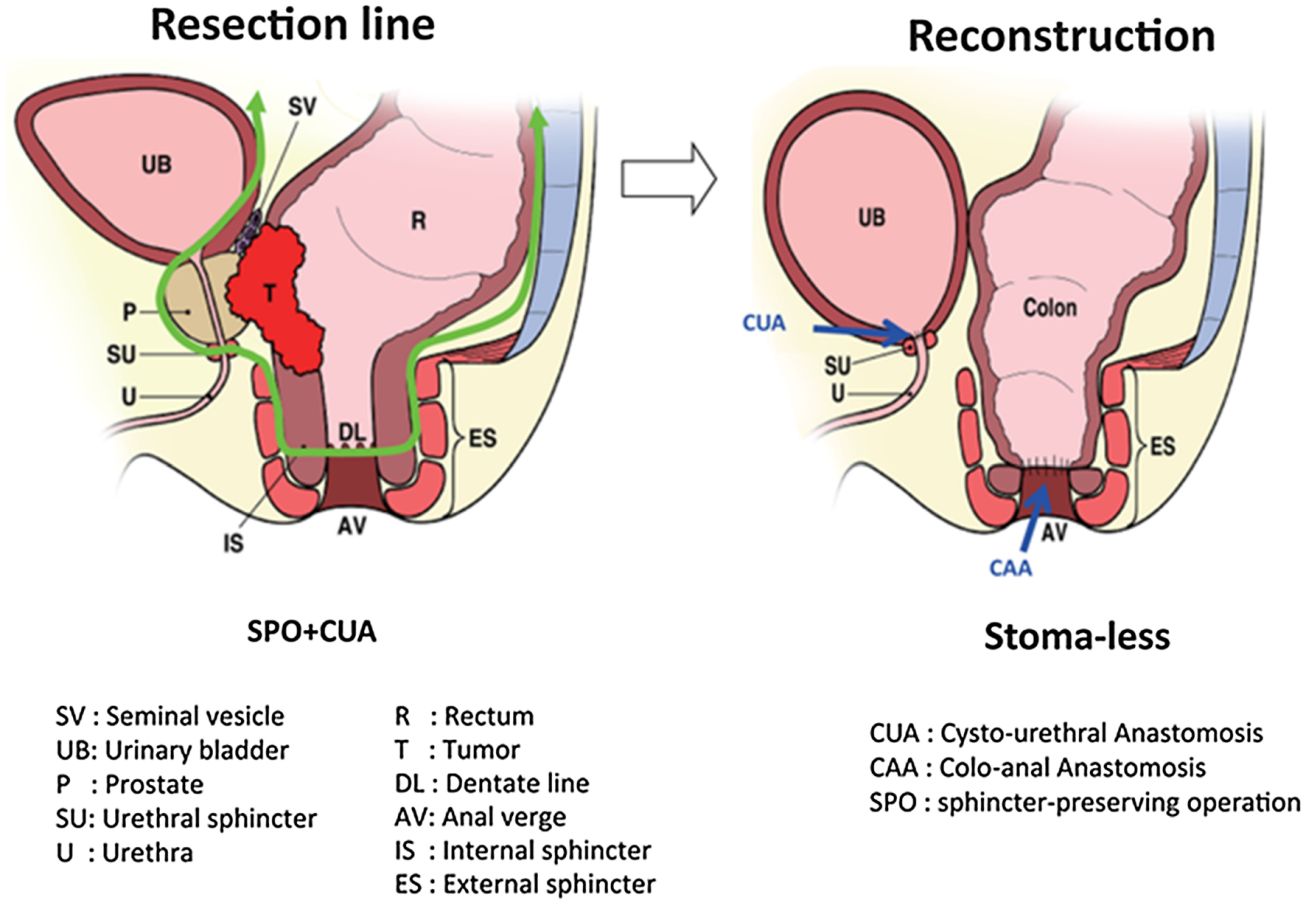
Bladder-sparing extended resection

**Fig. 2:**
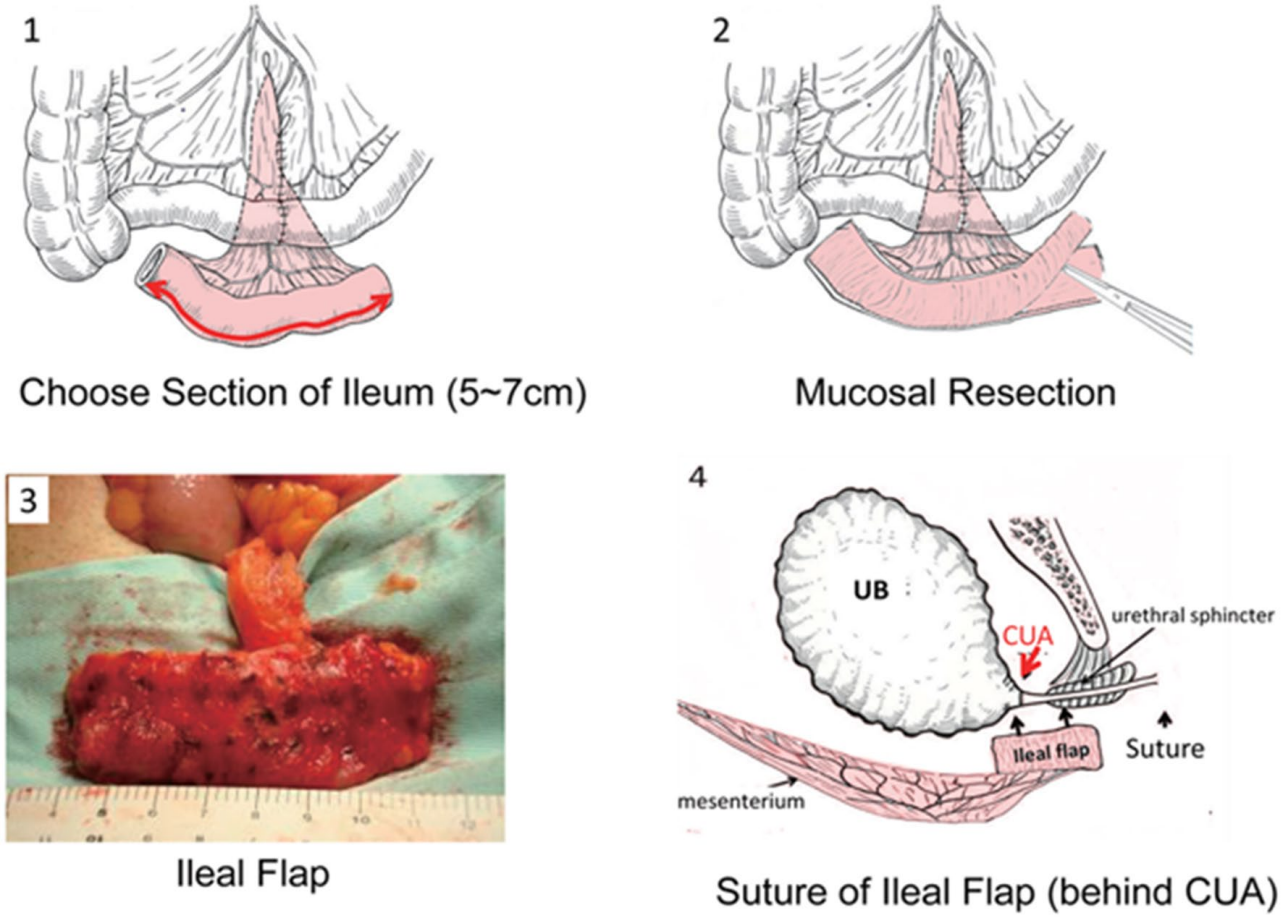
Procedure for the creation of an ileal flap

The surgical technique was as follows: after placing the patient in the lithotomy position, total mesorectal excision with lateral pelvic node dissection was performed (although lateral node dissection is not standard outside of Japan). The ureters were located and carefully protected throughout the procedure, and the superior vesical artery was preserved bi- or unilaterally. The status of involvement of the rectum and base of the bladder was investigated at this point. After confirming the absence of wide-ranging involvement of the urinary bladder, bladder sparing surgery was deemed possible. The rectal cancer, prostate, and seminal vesicles were resected en bloc cooperatively, by colorectal and urological surgeons using the usual methods for radical prostatectomy and ISR or APR. APR was performed when safe surgical margins could not be obtained by ISR. Frozen section examination of the resected specimen was done to confirm cancer-free radial and distal margins, evaluate the extent of pelvic invasion, and determine whether limited resection was possible. If tumor invasion was suspected in the surgical margins on the intraoperative histological examination, the operative procedure was converted to TPE. After confirming preservation of the membranous urethra and bladder, the bladder neck was reconstructed and a CUA was created by urological surgeons (Fig. 1). An ileal flap, made using 5 cm of mucoresected ileum, was placed with tissue surrounding the CUA (Fig. 2). The ileal flap has a good blood supply, which is why it was our first choice. When the omentum was sufficiently long, we considered adding an omental flap to the ileal flap. If the sphincter urethra muscle was sacrificed because of probable tumor invasion, a cystostomy was created for voiding through a catheter. Finally, a CAA was created with a diverting stoma or permanent colostomy by colorectal surgeons. We resected the prostate with a sufficient margin when it was thought that the rectal cancer had invaded the prostate before CRT. The bladder catheter was left in place for 2–3 weeks and removed after cystography had confirmed an intact anastomosis. The diverting stoma was closed 3 months after radical surgery.

### Evaluation of CUA leakage

On postoperative day 28, the strength of the CUA was assessed by cystography using 100–150 ml of standard contrast agent (sodium amidotrizoic acid meglumine injection, Urografin; Bayer) diluted in 100–150 ml of 0.9% NaCl inserted under gravity into the bladder via the Foley indwelling catheter. Anteroposterior and oblique views were obtained after contrast instillation. The cystography findings were evaluated by an experienced urologist. Perianastomosis leakage was classified as no leakage or leakage. In a patient with no leakage, the catheter was removed the same day. For a patient with leakage, radiological follow-up was conducted after 1–3 months. The leakage grade was classified using the National Cancer Institute Common Terminology Criteria for Adverse Events (CTCAE; urethral anastomotic leak) [21].

### Neoadjuvant therapy

Five patients (T4, N2: *n* = 3; T4, N1: *n* = 1; T4,N0: *n* = 1) between 2001 and 2010 agreed to undergo preoperative radiochemotherapy using our old protocol, despite this treatment for resectable rectal cancer not being standard in Japan, even for patients undergoing TPE. These patients received 45 Gy to the whole pelvis over a 5-week period, followed by resection after 2 weeks. In addition, 5-fluorouracil (5-FU) was administered as a continuous infusion at 250 mg/m^2^/day during the radiotherapy to enhance the radiotherapeutic efficacy. Postoperative chemotherapy (5-FU/leucovorin therapy or FOLFOX) was offered to patients with pathological stage III disease.

### Follow-up

The mean follow-up period was 70 months (range 0–162 months). Examinations were performed every 3 or 4 months for 2 years postoperatively, and every 6 months thereafter. These comprised clinical examinations; laboratory tests including tumor marker levels; lung, liver, and pelvic CT; and physiological assessment using anal manometry and uroflowmetry.

### Statistical analysis

The starting point for survival and recurrencefree intervals was the day of the operation, and data ofpatients who were alive or free of recurrence were censored at the last follow-up. OS was defined as the time from radical surgery until death from any cause. Local recurrence was defined as recurrence confined to the pelvis, and distant recurrence was defined as recurrence outside of the pelvis. Statistical analyses wereperformed using SPSS for Windows v. 11.0 J (SPSS Japan, Tokyo, Japan). OS and DFS curves were calculated using the Kaplan–Meier method.

### Ethical conduct of the study

The present study was registered with the University Hospital Medical Information Network Clinical Trials Registry (no. 000010530). The institutional review broad approved the protocol for this study. An independent safety monitoring committee monitored the safety of the patients throughout the study period. The study was performed under the Declaration of Helsinki and Japanese Good Clinical Practice Guidelines.

## Results

### Operation type

Twenty patients had localized tumors with clinical involvement of the prostate, three had clinical T3 lower rectal tumors with synchronous primary prostate cancer, and one underwent radical prostatectomy. Five patients had local recurrence with clinical involvement of the prostate. No procedures were converted to TPE because of inadequate margins. Twenty-two patients underwent anal SPOs (ISR, *n* = 18; U-LAR, *n* = 4) with radical prostatectomy, and 16 underwent APR with radical prostatectomy because the lower edge of the tumor was very close to the anal verge or the tumor involved the external anal sphincter. One patient underwent recurrent tumor resection and radical prostatectomy after APR.

Urinary reconstruction was performed with CUA in 32 patients, a neobladder was created in one patient, and cystostomy was performed in 6 patients because of the intraoperative histological probability of cancerous invasion of the sphincter urethra muscle. Five patients underwent an ileal flap operation, two underwent an omental flap operation, and three underwent an operation using both flaps. Twenty-two patients had no stoma, 11 had a single stoma, and 6 had a fecal stoma and cystostomy (Tables 1, 2). The median operative time was 505.5 min (range 315–857 min) and the median intraoperative blood loss was 3002 ml (range 225–12,531 ml).

**Table 2:** Type of surgery and pathological findings

	Primary, *n* = 34	Recurrence, *n* = 5
Type of surgery*	LAR (5.9%)	LAR (40.0%)
	APR (41.2%)	APR (40.0%)
	ISR (50.0%)	Recurrence tumor resection: 1 (20.0%)
	ISR + total cystectomy + neobladder: 1 (2.9%)	
Reconstruction	Urinary CUA: 28 (82.3%)	Urinary CUA: 4 (80.0%)
	CUA with flap: 9 (26.5%)	CUA with flap: 1 (20.0%)
	CS: 5 (14.7%)	CS: 1 (20.0%)
	Neobladder: 1 (2.9%)	
	Fecal CAA: 18 (52.9%)	Fecal DST: 2 (40.0%)
	DST (5.9%)	Stoma: 3 (60.0%)
	Stoma: 14 (41.1%)	
Depth of invasion (pT)	pT4: 12 (35.3%)	
	pT3: 15 (44.1%)	
	pT2: 4 (11.8%)	
	pT1: 1 (2.9%)	
	No tumor: 2 (5.9%)	
Involved organs	Prostate: 9 (26.5%)	Prostate: 1 (20.0%)
	Prostate + SV: 1 (2.9%)	Prostate + SV: 1 (20.0%)
	ES: 1 (2.9%)	SV: 2 (40.0%)
	Small intestine: 1 (2.9%)	
Node involvement (pN)	pN1: 12 (35.3%)	
	pN3: 6 (17.6%)	
Surgical margins	Negative, *n* = 34 (100%)	Negative, *n* = 5 (100%)

No stoma, *n* = 22; single stoma, *n* = 11; CS + stoma, *n* = 6

*ISR* intersphincteric resection, *APR* abdominoperineal resection, *CUA* cysto-urethral anastomosis, *CS* cystostomy, *CAA* colo-anal anastomosis, *ES* external anal sphincter

*All patients underwent radical prostatectomy

### Pathological findings

All resected margins were examined pathologically and confirmed to be cancer-free. The final pathological examination indicated pT4 in 16 patients (41.0%), with histological cancerous invasion of the prostate in 11; a pT3 tumor with fibrosis or inflammatory changes surrounding the tumor in 15; and a pT2 tumor with the same histological changes in 4. The T1b patient, 2 of the 4 pT2 patients, and 1 of the 15 pT3 patients had synchronous primary prostate cancer, and it was difficult to separate the rectal tumors safely from the prostate during the operation in these patients. Curative resection was achieved in 38 patients by bladder-preserving surgery and in one patient by using a neobladder.

### Morbidity and mortality

Table 3 summarizes the morbidity for this series. Seven patients had no postoperative complications. Of the 32 patients (82.1%) who had a complication, a CUA leak was identified in 17 (51.5%). A colo-anal anastomotic leak developed in 4 of 18 patients after an ISR and a double-stapling anastomosis leak developed in 1 of 4 patients after LAR. One patient with an anastomotic leak and ISR suffered from colo-anal anastomotic stenosis before closure of the diverting ileostomy. Additional surgery for the anastomotic stricture was thus performed by plastic surgeons, and the diverting stoma was closed 14 months after the initial operation. Other complications included pelvic abscess (10.3%, 4/39), wound infection (10.3%, 4/39), and bowel obstruction (17.9%, 7/39). There was no mortality in hospital or within 30 days postoperatively.

**Table 3:** Postoperative complications anastomotic leakage

Anastomotic leakage	SPO with CUA: 11/22 (50.0%)
	APR with CUA: 6/11 (54.5%)
	CUA leakage with flap: 3/10 (30.0%)
	CUA leakage with no flap 14/23 (60.9%)
	CAA: 4/18 (22.2%), DST:1/4 (25.0%)
Pelvic abscess	4/39 (10.3%)
Wound infection	4/39 (10.3%)
Ileus	7/39 (17.9%)
Overall morbidity	32/39 (82.1%)
Mortality	0/39 (0%)

*SPO* sphincter-preserving operation, *CUA* cysto-urethral anastomosis, *CAA* colo-anal anastomosis

### Leakage from the CUA

A CUA leak was identified in 17 patients and required catheterization through the anastomosis site for 8 to 108 weeks postoperatively. Of the 10 patients who had additional flap operations, 3 (30.0%) suffered leakage, whereas of the 23 patients who had no flap operation, 14 (60.9%) suffered leakage (Table 3). Two patients with ileal flaps and one with ileal and omental flaps suffered CUA leakage. The mean periods of catheterization in the patients with leakage were 31 weeks (8–108 weeks) for those without a flap and 16 weeks (8–20 weeks) for those with a flap. The period of urinary catheterization was more than 30 weeks in 4 of the 14 patients with leakage after surgery without a flap (33.3%; 2 patients without IF: periods of catheterization unknown), but in none of those with leakage after flap surgery (Table 4). Twelve patients with no flap and three patients with an additional flap had CTCAE grade 2 leakage. Two patients with no flap had grade 3 leakage and required reoperation using a gracilis muscle flap, whereas none of those with leakage after additional flap surgery required reoperation.

**Table 4:** Duration of catheterization in the cystourethral anastomosis leakage group

Ileal flap (IF)	Number of patient	Duration (weeks) Mean (range)	More than 30 weeks with catheterization (*n*)	CTCAE Grade3 (*n*)
Without IF	12	31 (8–108)	4 (33.3%)	2 (16.7%)
With IF	3	16 (8–20)	0 (0%)	0 (0%)

Two patients without a flap had unknown periods of catheterization

### Survival

By the last follow-up, in January 2013, 28 patients were alive and 11 had died. The causes of death included multiple bone metastases (*n* = 1), multiple lung metastases (*n* = 4), multiple liver and bone metastases (*n* = 1), multiple lung and bone metastases (*n* = 1), and local re-recurrence (*n* = 2). The patient treated with a neobladder died of an abdominal injury sustained in a fall (Table 5). The estimated 5-year OS and DFS rates were 68.6 and 51.0% for patients with primary disease and 40.0 and 20.0% for those with recurrence, respectively.

**Table 5:** Patients with recurrence and re-recurrence

	Primary, *n* = 15	Recurrence, *n* = 5
Lung	3	
Liver	5	1 (liver resection → ANED)
Bone	1	
Lung + bone	1	
Local	1 (paraanastomotic site tumor resection → ANED)	2 (death, Local resection → death)
Liver + lung + local	2 (liver resection → lung and local → chemotherapy → death, liver resection → lung resection → left obturator internus muscle recurrence → Cape + RT → CR → ANED)	
Liver + bone	1	
Lung + local	1 (lung → chemotherapy → local resection → death)	
Lymph		1 (chemotherapy → ANED)
Liver + abdominal wall		1 (local resection → ANED)

*ANED* alive with no evidence of disease, *AWD* alive with disease

### Local and overall recurrence

Recurrence developed in 15 (44.1%) of the 34 patients with primary disease, and the incidence of local recurrence was 26.7% (Table 5). Local recurrences developed in the presacral area in two patients, in the perianastomotic site of the CUA in one, and in the left obturator internus muscle in one. One patient with recurrence in the presacral area and one with recurrence in the perianastomotic site of the CUA underwent tumor resection with sufficient surgical margins. The other patient with recurrence in the presacral area had simultaneous lung metastasis and received chemotherapy. The patient with recurrence in the left obturator internus muscle received chemoradiotherapy and complete remission was achieved (Table 5). This patient and the patient with recurrence in the preanastomotic site had no evidence of disease after resection of the local recurrence.

## Discussion

Orthotopic neobladder surgery is often used as an alternative for patients undergoing radical cystectoprostatectomy for bladder cancer to enable voiding via the urethra with urinary continence [16, 17]. Patients undergoing radical prostatectomy with CUA for carcinoma of the prostate can also void with continence via the urethra. SPOs for lower rectal cancer have become more common with improved surgical techniques such as ISR, which was devised in the 1980s, and further concepts established in the 1990s.

ISR is a procedure that gives sufficient surgical margins by removing part or all of the internal anal sphincter and restoring bowel continuity for patients with rectal cancer located within 5 cm of the anal verge. Fixation of the lower urinary tract organs, such as the prostate, in primary locally advanced rectal cancer is not uncommon in men. Standard therapy for such patients in the absence of extra-pelvic metastases has been total pelvic exenteration to ensure negative surgical margins. However, this procedure requires urinary and fecal diversion through the formation of double stomas. In 1966, separate prostatectomy was suggested as a routine additional procedure to prevent voiding problems after APR for rectal cancer. Three cases of patients with synchronous rectal and prostate cancer, who underwent separate dissections were reported recently [12]. Another report described combined radical retropubic prostatectomy and proctosigmoidectomy en bloc in 11 selected patients with cancer limited to the prostate [13–15, 18].

We believe that even more limited excision is feasible and preferable if the tumor can be removed en bloc. Preservation of the prostate may also be possible if significant tumor shrinkage can be achieved by neoadjuvant therapy such as preoperative chemoradiation or chemotherapy. However, preoperative chemoradiotherapy for advanced rectal cancer is not a standard option in Japan, even for patients undergoing TPE, and it may also cause postoperative complications. Since 2001, we have used a combined approach for primary or recurrent rectal cancer involving lower urinary tract organs or with synchronous prostatic cancer. In our series of 39 patients treated during this period, the bladder was preserved in 38 patients to avoid TPE, and anal SPOs using ISR techniques were performed whenever possible. Cancer-free margins were obtained in all patients.

Similar procedures without ISR were first described by Campbell et al. [13] in a report of two patients in whom en bloc excision yielded negative surgical margins. Wiig et al. [18] found no local recurrence in follow-up for 10–50 months after en bloc radical prostatectomy for 6 patients with locally advanced or recurrent rectal cancer involving the prostate, with 4 of 5 patients with a CUA having a good quality of life and none wanting an ileal conduit.

There have been a few reports on these operations of bladder preservation, but the complication rate of leakage from the CUA was relatively high. In the present series, anastomotic urethral leak developed in 17 (53.1%) of 32 patients with a CUA. Similarly, Wiig et al. [18] found that 3 (50%) of 6 patients with a CUA had an anastomotic urethral leak with one major leakage. The frequency of anastomotic leaks was significantly higher in patients undergoing APR with a CUA. In contrast, the urethral anastomotic leakage rate was lower in patients undergoing ISR or ultra-LAR with a CUA, as the neorectum is located behind the CUA. In studies of the leakage rate from a CUA in patients undergoing radical retropubic prostatectomy only [22, 23], extraversion was not found in 135 (75%) of 179 cystograms, and the clinically prolonged leakage rate was 0.6% (11/1796). All these patients had the rectum behind the CUA. Urethral anastomotic leaks are thus probably due to a lack of supporting tissue behind the anastomosis after removal of the rectum in patients who have undergone APR. An additional flap operation may decrease leakage from a CUA and reduce the duration of urinary catheterization for a patient with leakage. By performing this new ileal flap operation we improved the leakage rate to 30.0% (3/10) from 60.7% (14/23). Four of the 16 patients with leakage after surgery without a flap (33.3%) required urinary catheterization for more than 30 weeks, whereas none of the patients with leakage after flap surgery required prolonged urinary catheterization. This demonstrates the effectiveness of this method and how it improves quality of life. Measures to prevent urethral anastomotic leakage using a flap of the greater omentum behind the anastomosis or an additional ileal flap, such as a gracilis flap, thus appear warranted.

The method described in this study is a part of an operation for cancer; therefore, prognosis and recurrence are very important considerations. Similar procedures were described by Campbell et al. [13] in two patients who had no evidence of local recurrence at their 1-year follow-up. Wiig et al. [18] also found no local recurrence during follow-up for 10 to 50 months after en bloc radical prostatectomy in 6 patients with locally advanced or recurrent rectal cancer involving the prostate. However, as there were few cases of long-term follow-up included in these reports [13, 18], the long-term outcomes of the procedures are unclear.

In the present study, six patients (15.3%) suffered local recurrence during follow-up, including one recurrence in the presacral area. This local recurrence was unlikely to have been prevented had the patient undergone TPE. The patient underwent successful resection of the recurrent tumor, but died of multiple lung metastases 86 months after the initial operation. In another patient, local recurrence developed at the paravesical site. This patient also underwent resection of the recurrent tumor and remains re-recurrence free after 90 months. In a third patient, local recurrence developed at the parasacral site. Resection of the recurrent tumor was not possible and despite chemotherapy, the patient died 38 months after the first operation. In a fourth patient, local recurrence developed in the left internal obturator muscle. Again, tumor resection was not possible, but chemoradiotherapy was given and this lesion is in complete remission, with the patient being re-recurrence free after 60 months. The last two patients both had recurrent lesions in the pelvic area at their first operation in our hospital. One underwent resection of the recurrent tumor, but died 55 months after the operation; and for the other patient, resection of the recurrent tumor was not possible and he died 1 month after the operation.

In 2013, Yang et al. published a systematic review on pelvic exenteration for rectal cancer [24]. The 5-year survival rate for patients with primary advanced rectal cancer ranged from 31 to 77% (median, 52%) [25–40] in 17 studies reviewed, whereas that for patients with locally recurrent rectal cancer ranged from 0 to 37% (median, 18%) in 13 studies reviewed [25, 27, 30, 31, 34–36, 38–43]. Moreover, the 5-year disease-free survival rate was 52 and 13% for those with primary vs. recurrent tumors, respectively [34]. In the current study, acceptable 5-year OS and DFS rates of 68.8 and 51.1%, respectively, were achieved for patients undergoing surgery for primary locally advanced very low-lying rectal cancer, with a mean follow-up of 70 months (range 0–162 months). For those with recurrence, the 5-year OS was 40.0% and the 5-year DFS was 20.2%. Despite our concerns about the risk of local recurrence after limited excision to preserve the urinary bladder, the local recurrence rate in this series was relatively low. A remaining problem is prevention of distant metastases, but to date, the procedures reported here appear to be oncologically safe for selected patients with rectal cancer involving the prostate.

Patients with a CUA also reported satisfactory control of voiding function, with a voiding style resembling that of patients with an ileal neobladder. Unfortunately, six patients required cystostomy because the sphincter urethra muscle was sacrificed due to probable cancerous invasion. These patients voided via an inserted catheter that was exchanged once a month, much like those with an ileal conduit. An obvious difference between neobladder and bladder-sparing surgery is that the neobladder is made using intestine, which results in inevitable long-term complications such as mucus production, nutritional abnormalities, metabolic acidosis, skeletal demineralization, and a risk of malignant transformation in the intestinal segment [44, 45]. No such problems are associated with bladder-sparing surgery.

In conclusion, this prospective study demonstrates the effectiveness of the ileal flap method for reducing CUA leakage and improving quality of life. Our results suggest that this new method should be evaluated using a similar trial design for patients with advanced rectal patients, to avoid colostomy.
